# The Potential Role of Microbial Biostimulants in the Amelioration of Climate Change-Associated Abiotic Stresses on Crops

**DOI:** 10.3389/fmicb.2021.829099

**Published:** 2022-01-14

**Authors:** Ayomide Emmanuel Fadiji, Olubukola Oluranti Babalola, Gustavo Santoyo, Michele Perazzolli

**Affiliations:** ^1^Food Security and Safety Niche, Faculty of Natural and Agricultural Sciences, North-West University, Potchefstroom, South Africa; ^2^Instituto de Investigaciones Químico Biológicas, Universidad Michoacana de San Nicolás de Hidalgo, Morelia, Mexico; ^3^Center Agriculture Food Environment (C3A), University of Trento, San Michele all’Adige, Italy; ^4^Research and Innovation Centre, Fondazione Edmund Mach, San Michele all’Adige, Italy

**Keywords:** arbuscular mycorrhizal fungi, plant-microbe interaction, PGPR, sustainable agriculture, climate change

## Abstract

Crop plants are more often exposed to abiotic stresses in the current age of fast-evolving climate change. This includes exposure to extreme and unpredictable changes in climatic conditions, phytosanitary hazards, and cultivation conditions, which results in drastic losses in worldwide agricultural productions. Plants coexist with microbial symbionts, some of which play key roles in the ecosystem and plant processes. The application of microbial biostimulants, which take advantage of symbiotic relationships, is a long-term strategy for improving plant productivity and performance, even in the face of climate change-associated stresses. Beneficial filamentous fungi, yeasts, and bacteria are examples of microbial biostimulants, which can boost the growth, yield, nutrition and stress tolerance in plants. This paper highlights recent information about the role of microbial biostimulants and their potential application in mitigating the abiotic stresses occurring on crop plants due to climate change. A critical evaluation for their efficient use under diverse climatic conditions is also made. Currently, accessible products generally improve cultural conditions, but their action mechanisms are mostly unknown, and their benefits are frequently inconsistent. Thus, further studies that could lead to the more precisely targeted products are discussed.

## Introduction

Global climate records in the last decades have revealed a rise in global temperature alongside changes in rainfalls, resulting in various serious implications on environmental and agricultural aspects (Füssel et al., 2012). Crop plants are more frequently exposed to abiotic stresses caused by climate change because, aside from direct implications of abiotic stresses on plants, climate change could increase the number of pests and diseases, as well as increase the severity and frequency of the outbreak of diseases (De Wolf and Isard, 2007; Garrett et al., 2016). According to recent estimates, abiotic stresses are anticipated to cause up to 50% losses, or higher, in worldwide agricultural productivity, depending on the region (Kumar and Verma, 2018). These losses, coupled with the continual rise in the human population, revealed that about 60% boosting of agricultural production is needed to meet the world food needs (Wild, 2003), with a concrete risk of dramatic deforestation increment and loss of natural ecosystems (Byerlee et al., 2014). Increased plant resilience to mitigate climate change-associated stresses is a sustainable method for ensuring food security with a restricted increase in agricultural surface, and the use of microbial biostimulants is one of the best options to achieve this goal (Calvo et al., 2014; Yakhin et al., 2017).

Plants are associated with a diverse group of microorganisms (the microbiome) in their endosphere (internal compartments), rhizosphere (attached soil to roots), and phyllosphere (aboveground parts), making microbial biostimulants particularly fascinating (Compant et al., 2019; Babalola et al., 2020). Crop plants coexist with microbial symbionts, which play key roles in plant production, performance, nutrition, and tolerance to abiotic stress (Vandenkoornhuyse et al., 2015; Enebe and Babalola, 2018; Ojuederie et al., 2019). For instance, geological evidence indicates that the relationship between microbes and plants predates the emergence from the water, suggesting that symbiosis involving arbuscular mycorrhizal was important in the process of terrestrialization (Selosse and Le Tacon, 1998). Moreover, microorganisms are involved in multiple biogeochemical cycles, such as nitrogen and carbon cycling, nitrogen fixation, soil formation and plant nutrition acquisition in the ecosystems (Wagg et al., 2014; Igiehon et al., 2019). As a result, many microbial symbionts can be used as a biofertilizer, releasing additional nutrients to the plant through synergistic mechanisms, which include nitrogen fixation (e.g., *Mesorhizobium loti*, *Rhizobium etli*, *Azotobacter vinelandii*, and *Azospirillum brasilense*), phosphate solubilization (e.g., Arbuscular mycorrhizal fungi, *Azospirillum* spp., *Bacillus* spp., and *Pseudomonas* spp.), cellulolytic activity (*Aspergillus* spp., *Trichoderma* spp., *Bacillus* spp., and *Penicillium* spp.), soil acidification (*Bacillus* spp. and *Arthrobacter* spp.), and production of siderophores (e.g., *Pseudomonas* spp.) (Bhattacharyya and Jha, 2012; Orozco-Mosqueda et al., 2021). Also, thanks to its abilities to boost plant development, defenses, antibacterial compounds, combat pathogen infections and feed on nematodes, *Trichoderma* spp. is a well-studied symbiotic fungal genus (Adnan et al., 2019; Szczałba et al., 2019). Despite the beneficial effects exhibited on their hosts, some of which include increased protection from abiotic stresses and nutritional efficiency, some weaknesses may limit the use of *Trichoderma* spp. as commercial biostimulant products, such as difficulties of *in vitro* cultivation and escalating of bioproduction, the lack of understanding on host specificity and population dynamics in the agroecosystem (Du Jardin, 2015). Other types of fungi can form part of the beneficial microbiome associated with plants, such as yeasts belonging to *Brettanomyces naardensis*, *Candida oleophila*, *Aureobasidium pullulans*, *Metschnikowia fructicola*, *Cryptococcus albidus*, and *Saccharomyces cerevisiae* (Freimoser et al., 2019; Nafady et al., 2019). Foliar infections can be controlled by yeasts that could colonize the leaf of a plant using direct antagonism (Preininger et al., 2018) or through the induction of systemic resistance (Lee et al., 2017). Likewise, yeast inhabiting the soil can enhance the growth of the plant through phosphate solubilization, digestion of organic materials, soil aggregation and stimulation of root development, and suppressing root infections (Sarabia et al., 2018). Plant growth-promoting bacteria (PGPB), which includes rhizobacteria or bacterial endophytes, are known to majorly populate the plant rhizosphere and the most studies genera are *Azospirillum*, *Azotobacter*, *Arthrobacter*, *Burkholderia*, *Gluconacetobacter*, *Pseudomonas*, *Bacillus*, *Streptomyces*, and *Serratia* (Kour et al., 2020a). Additional genera are more recently proposed as possible bioinoculants with biocontrol and/or plant growth-promoting activities, such as *Rouxiella badensis* and *Rahnella* spp. (Ulloa-Muñoz et al., 2020; Morales-Cedeńo et al., 2021). Physiological, molecular, and biochemical investigations of the interactions that exist between plant and beneficial microorganisms have shown that the presence of microbe-induced plant stress responses (Farrar et al., 2014; Igiehon et al., 2021; Igiehon and Babalola, 2021), which may trigger induced systemic tolerance (IST) against abiotic stressors (Yang et al., 2009; Vacheron et al., 2015).

Microbial biostimulants are a viable alternative for supporting plants exposed to abiotic stresses in the current context of fast-developing climate change (Santoyo et al., 2021b). While recent advancements and laboratory studies have revealed the positive activities of plant-associated microorganisms, the efficacy of microbial biostimulants is yet to be successfully validated in field experiments. As a result, microbial biostimulants are often used as supplemental therapies rather than being used to their full potential in crop management. The goal of this paper is to summarize current information about microbial biostimulants, especially the current commercially available products, examine their applications in enhancing plant tolerance to abiotic stresses caused by climate changes, and forecast the creation of novel products that may be used in adverse conditions. Also, limitations in the application of microbial biostimulants under field conditions alongside further studies required for the better development of targeted products are discussed.

## Climate Change and Its Impact on Agriculture

Climate change is caused by a variety of factors, including variations in solar radiation, shifts in the Earth orbit, changes in the composition of the atmospheric gases, ocean oscillations, and changes in the surface characteristics of the soil (Meena et al., 2017; Cassia et al., 2018). The impacts of climate change, which often influence not only human existence but also ecological systems, elicit strong conflicts and emotions (Rising and Devineni, 2020). Agriculture is highly vulnerable to climate change because it is so reliant on soil quality, irrigation and weather conditions (Kumar et al., 2021). Floods, droughts, heat stress, rainfall unpredictability, and severe weather occurrences have a significant negative influence on global agricultural practices as a result of climate change (Myers et al., 2017; Rising and Devineni, 2020; Fiodor et al., 2021). It is clear that climate change may have a greater impact in some regions of the world, suggesting that existing farming systems and infrastructure will need to change and rapidly adapt to future changes (Müller et al., 2011). Plants, as stationary creatures, are compelled to adapt to their surrounding conditions to live and they can respond to diverse abiotic stressors with precise acclimations of physiological, developmental, and metabolic processes (Meena et al., 2017; Myers et al., 2017). Unfortunately, plant tolerance to one abiotic factor may be reduced as a result of exposure to another abiotic or biotic factor, increasing plant sensitivity (Laanisto and Niinemets, 2015). However, a multivariate acclimation in response to multiple abiotic factors is also possible (Laanisto and Niinemets, 2015).

### Global Warming

The Intergovernmental Panel on Climate Change (IPCC) defines global warming as an “increase in both sea surface temperature and surface air averaged above 30 years” throughout the planet (Allen et al., 2018). It is also claimed that as the Earth moves from the relatively stable Holocene to the more dynamic Anthropocene, where human activities are now implicated as a major source of climate change and human-caused global warming has now surpassed pre-industrial levels by roughly 1^°^C (Allen et al., 2018). Subsequently, several parts of the world have experienced a far greater rise in temperature than the worldwide average, leading to a possible rise from 1.5 to 4.8^°^C by 2100, according to estimates (Fiodor et al., 2021; Jagadish et al., 2021). Furthermore, land-based warming is outpacing the global average and it was estimated 1.5^°^C in the years 2006–2015 compared to the years 1850–1900 (Fiodor et al., 2021).

Temperature is an important environmental factor that affects physiological processes and plant growth. Temperature increases promote quicker mass growth and shorter culture times (Zhao et al., 2017). However, a sudden increase or decrease in temperature can damage plant cells, resulting in reduced growth and yield overall (Zhao et al., 2017; Seguini et al., 2019). Also, for every 1^°^C increase in world average temperature, 3.1 to 7.4% yield losses were estimated in rice, maize, soybean and wheat for over 29 years of warming trends (Seguini et al., 2019). Another research revealed a 4 and 6% reduction in maize and wheat yields, respectively (Lobell and Gourdji, 2012). Heat stress is a limiting factor for photosynthesis in C3 crops, like rice and wheat, as well as C4 plants, like maize and sugarcane crops (Crafts-Brandner and Salvucci, 2002; Song et al., 2014; Zhao et al., 2017). Plants are also affected by temperature rise due to changes in humidity since reduced water vapor concentration in the air causes water loss from the plant, forcing stomata to be closed and lowering photosynthetic efficiency and leaf cooling by transpiration (Zhang et al., 2019; Díaz-Barradas et al., 2020). Moreover, water stress in plants is caused by prolonged high temperatures, which results in water shortages (Díaz-Barradas et al., 2020).

The hazard of global warming to crops is not limited to changes in global mean temperature, but also to sea-level rise, desertification, extreme weather conditions and changed precipitation (Russo et al., 2014). For example, due to water mixing, sea-level rise causes salinization of fresh water and land loss (Fiodor et al., 2021). Exacerbation of uncommon weather phenomena, such as floods, heatwaves, and severe precipitation, is being reported when the hydrological cycle accelerates with global warming (Hutchins et al., 2019; Perkins-Kirkpatrick and Lewis, 2020; Hawkings et al., 2021). Thus, global warming might have a slew of unanticipated consequences. For example, the melting of Greenland’s ice sheet is supposed to become a possible source of hazardous mercury contamination in the Atlantic seas (Hawkings et al., 2021).

### Greenhouse Gas Emission

Greenhouse gases (GHGs) are parts of the troposphere of the Earth, and they may sustain solar energy. Thanks to GHGs, the average temperature in most areas remains below 40°C, allowing plants to flourish freely (Fiodor et al., 2021). Forest fires, seas, earthquakes, permafrost, wetlands, and volcanoes are the natural environmental sources of GHG emissions that are generally absorbed by the environment (Yue and Gao, 2018). However, human activity causes an excess in GHG emission, which upsets the equilibrium (Yue and Gao, 2018). Methane, nitrous oxide and carbon dioxide are examples of the three most common greenhouse gases generated by human activity and more than 90% of human-derived global warming is thought to be caused by these gases (Wu and Mu, 2019). Along with the rise in greenhouse gases release, global warming processes have become more intense as a result of urbanization, globalization, and industrialization (Yusuf et al., 2020), while the ever-increasing human population has increased the need for energy production.

When comparing carbon dioxide levels in the atmosphere before and after the industrial revolution, it is apparent to state that human activities are the primary source of this gas (Meena et al., 2017; Hawkings et al., 2021). Carbon dioxide levels are quickly rising, from 280 ppm before the industrial revolution to 400 ppm now and it is estimated that 540 ppm will be achieved by 2100 (Myers et al., 2017). Deforestation-related increases in land use account for one-fifth of total carbon dioxide emissions (Szczałba et al., 2019). Over the decades, vast amounts of carbon dioxide are introduced into the atmosphere as fossil carbon that has accumulated in the Earth’s crust over millions of years (Anwar et al., 2020). Greenhouse gas emissions in the European Union (EU) and United States of America (USA) were 4.4 and 6.5 billion metric tons of carbon dioxide equivalent in 2018–2019, respectively (Fiodor et al., 2021; Gołasa et al., 2021). China topped the list of the biggest polluters in 2014, accounting for 30% of all carbon dioxide emissions, while Japan, Russia, India, European Union and the United States contributed 4, 5, 7, 9, and 15% of carbon dioxide emissions, respectively (Fiodor et al., 2021). Different major economic sectors are associated to GHG emission, such as (i) industry, (ii) heat and electricity production, (iii) forestry, agriculture, and other land use, (iv) building, and (v) transportation (EPA, 2021). In particular, forestry, agriculture, and other land uses were responsible for 24% of worldwide greenhouse gas emissions in the year 2010, while only agriculture showed a proportion that ranged from 1 to 14%, which is similar to the production from the industry (Fiodor et al., 2021). Since 1990, greenhouse gas emissions in agriculture have risen by 12%, due to a 9% increase in nitrous oxide emissions from soil management practices and a 60% rise in nitrous oxide and methane emissions from animal manure management systems (EPA, 2021; Fiodor et al., 2021), indicating the increasing application of emission-intensive liquid systems over the years. However, agricultural soil management strategies that increase nitrogen availability in the soil and reduce nitrous oxide emissions are available (IPCC, 2014). Nitrous oxide emissions from agricultural areas are caused by the application of organic and chemical fertilizers, the growth of nitrogen-fixing crops, irrigation methods and organic soil draining (EPA, 2021). In particular, excess nitrogen application can result in increased nitrous oxide emission without contributing to the growth and yield of the crop, indicating that more precise nitrogen fertilization for optimum crop production may help lower greenhouse gas emissions (Fiodor et al., 2021).

Changes in nutrient composition and photosynthetic rates in plants are two major effects of GHGs in agriculture (Myers et al., 2017). Reduced transpiration occurs as a result of increment in carbon dioxide concentrations, with a consequent increment in photosynthesis and water use efficiency. Rapid plant growth in high carbon dioxide environments changes the composition of essential elements for animals and humans. For example, legumes and cereals grown under carbon dioxide concentration of 550 ppm have iron and zinc levels that are 3–11% lower compared to a plant grown under normal atmospheric concentration (Gornall et al., 2010; Sangiorgio et al., 2020). Likewise, phosphorus, calcium, potassium, zinc, sulfur, iron, copper, and magnesium concentrations decrease by about 5–13% in various crops when the carbon dioxide concentration is 690 ppm (Loladze, 2014). Under elevated carbon dioxide, a decrease in nitrogen and protein content in edible parts of C3 plants have been widely reported (Jobe et al., 2020), indicating an increased risk of nutritional problems for people living in the underdeveloped parts of the world (Loladze, 2014).

## The Impact of Microbial Biostimulants in Ameliorating Climate Change-Induced Stresses on Plants

### High Temperature

High temperatures affect the physiology of the plant by raising the respiration rates and leaf transpiration and altering photosynthate allocation (Munns, 2002; Malhotra, 2017). Rubisco affinity for carbon dioxide decreases at high temperatures and its affinity for oxygen increases (Jordan and Ogren, 1984; Sangiorgio et al., 2020). Carbon dioxide solubility is reduced more than oxygen when the temperature rises, lowering carbon dioxide concentration in the chloroplast in comparison to oxygen (Ku and Edwards, 1977). Furthermore, plants shut their stomata to decrease evapotranspiration water losses when the temperature rises. When stomata closure occurs, carbon dioxide concentration declines rapidly, but oxygen concentration rises, limiting photosynthesis, increasing the photorespiration (Bhattacharya, 2019).

Higher temperatures in spring would allow many cycles per year for annual crops, such as tomato (Sangiorgio et al., 2020) and lettuce (Pearson et al., 1997), enhancing early flowering in horticultural crops (Wheeler et al., 1993; Bisbis et al., 2018). On the other hand, increasing temperatures may provide difficulty for floral differentiation in several horticultural crops. For example, high temperatures enhance male floral development but reduce feminine flower differentiation in cucumbers (Sangiorgio et al., 2020). In fruit crops, such as apple (Funes et al., 2016), peach, and plum (Hazarika, 2013), difficulty in meeting cold requirements for floral differentiation might reduce crop production (Luedeling, 2012). In the long run, climate change may move fruit cultivation areas to the North (Luedeling, 2012), but moderate winter temperatures are capable of stimulating early blooming, thereby exposing plants to late frost risks (Vitasse and Rebetez, 2018). Finally, increasing temperatures may affect agricultural practice in tropical environments which often result in the extinction of vulnerable crops (Gornall et al., 2010).

**FIGURE 1:**
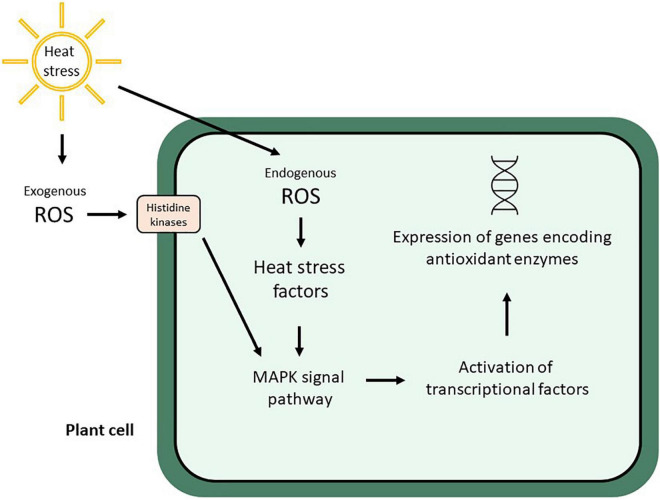
Plant cell response to exogenous and endogenous ROS production stimulated by heat stress (modified from Qu et al., 2013).

Heat stress causes complex molecular, biochemical, and physiological reactions in plants (Sangiorgio et al., 2020), which may result in the synthesis of heat shock proteins, enzymes that degrade reactive oxygen species (ROS), osmoprotectant chemicals, amino acids, sugars, and sulfur compounds (Shulaev et al., 2008). Thus, oxidative stress and ROS production are stimulated by heat stress, which is sensed by histidine kinases and Heat stress factors (Hsfs), then redox-sensitive transcription factors are downstream activated by the MAPK signal pathway, which subsequently activates other transcription factors (e.g., BF1c and Rboh) to turn on the expression of genes involved in the synthesis of antioxidant enzymes (Qu et al., 2013; Figure 1).

Hormonal signaling is in charge of heat stress responses. Ethylene is one among them (Byerlee et al., 2014), and it is involved not only in senescence, development, plant physiology, and development but also in plant responses to abiotic stressors, such as heat, salinity and drought (Dubois et al., 2018). Plant responses to heat stress can be boosted by microbial biostimulants. For example, the synthesis of ROS-degrading enzymes (e.g., superoxide, catalase, peroxidases, and dismutase) enhance heat stress tolerance and it can be boosted in plants colonized by beneficial bacteria, such as *Pseudomonas* and *Bacillus*, and mycorrhizal fungi, such as *Septoglomus deserticola* and *Septoglomus constrictum* (Duc et al., 2018). SoilPro^®^ (Liventia, TX, United States) is a microbial biostimulant that helps in improving soil quality and heat stress tolerance and it contains *P. fluorescens* and *P. aeruginosa*, which are commercialized for bioremediation, phytostimulation, and improved capabilities of soil fertility (Sangiorgio et al., 2020). Likewise, *Bacillus* spp. have been developed not only as biopesticides (e.g., *Bacillus amyloliquefaciens*, *Bacillus firmus*, *Bacillus pumilus*, *Bacillus thuringensis*, *Bacillus licheniformis*, *Bacillus sphaericus*, and *Bacillus subtilis*), and some biostimulant products made up of *Bacillus* spp. are available, such as Endox^®^ (Scam, Spa, Italy) and Activate^®^ (Natural resources Group, Inc., United States). While these microorganisms are used in many commercial treatments, whether alone or as a mixture, mitigation from heat stress is not usually stated as one of their benefits.

**FIGURE 2:**
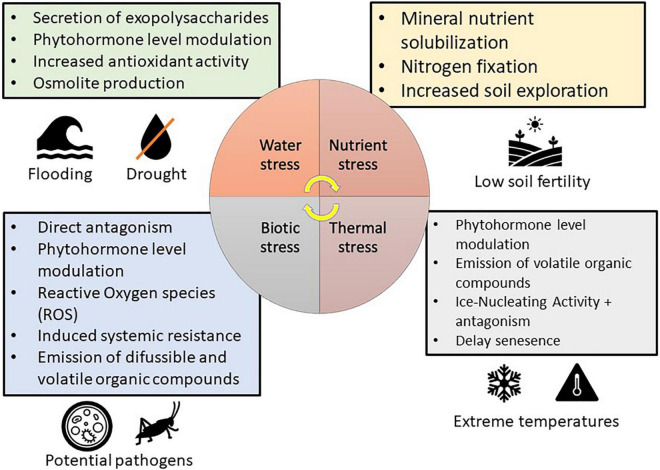
Protective mechanisms used by microbial biostimulants in mitigation of various stresses in plants (modified from Sangiorgio et al., 2020).

The use of microorganisms that limit ethylene emissions has a lot of promise because lowering ethylene levels in stressful situations might help plants escape the harmful effects of heat stress (Glick, 2014). In particular, bacteria that exhibit 1-aminocyclopropane-1-carboxylic acid (ACC) deaminase activity, appear to be highly promising, such as *Bacillus subtilis* BERA 7, *Leclercia adecarboxylata* MO1, *Pseudomonas fluorescens* YsS6, and *Pseudomonas migulae* 8R6 (del Carmen Orozco-Mosqueda et al., 2020). For example, the ACC deaminase-producing *Paraburkholderia phytofirmans* PsJN allows normal tomato development under heat stress (Esmaeel et al., 2018). Despite its apparent beneficial properties, *P. phytofirmans* PsJN is yet to be used as a commercial product (Sangiorgio et al., 2020). The ability of microbial biostimulants able to mitigate heat stress in crops was associated to the reduction of ROS content, the increase in heat shock protein and osmoprotectant (e.g., sugar and proline) content and the modulation of hormonal levels (Figure 2), such as ethylene and abscisic acid (Table 1). In particular, *Pseudomonas* sp. AKMP6 and *P. putida* AKMP7, *Glomus* sp., *B. aryabhatthai* SRBO2, *B. amyloliquefaciens*, *A. brasilense*, and *P. phytofirmans* (Ali et al., 2009; Ali and Xie, 2020). However, further efforts on the understanding of the mechanism of action of microbial biostimulants against heat stress and on the development of efficient formulations are required to mitigate the impact of heat stress on agricultural and food production by sustainable approaches.

**TABLE 1:** Microbial biostimulants that mitigate extreme temperature stress in crops.

Crop Plant	Microbes involved	Mechanism of action	References
**Protection against cold and freezing stress**			
Grapevine	*Paraburkholderia phytofirmans*	Production of 1-Aminocyclopropane-1-carboxylic Acid (ACC)	Theocharis et al., 2012
Pear and apple	*Pseudomonas fluorescens* A506	Competition with bacteria having INA**^+^**	Lindow and Brandl, 2003
Bean plant	*Pseudomonas fragi*, *Pseudomonas fluorescens*, *Pseudomonas proteolytica*, *Brevibacterium frigoritolerans*, and *Pseudomonas chlororaphis*	Reduction of reactive Oxygen Species (ROS) and lipid peroxidation	Tiryaki et al., 2019
Wheat	*Pantoea dispersa* 1A, *Pseudomonas* spp. and *S. marcescens* SRM	Production of ACC and IAA	Mishra et al., 2008; Selvakumar et al., 2008a,b
**Protection against heat stress**			
Sorghum and wheat	*Pseudomonas* sp. AKMP6 and *Pseudomonas putida* AKMP7	Reduction of reactive Oxygen Species (ROS), increment in content sugar, proline, starch, chlorophyll, protein, and amino acid, phytohormone production.	Ali et al., 2009, 2011
Tomato	*Glomus* sp.	Higher scavenging activity of ROS in the rots and leaves and reduction in peroxidation of lipid.	Duc et al., 2018
Soybean	*Bacillus aryabhatthai* SRBO2	Production of abscisic acid	Park et al., 2017
Wheat	*Bacillus amyloliquefaciens*, and *Azospirillum brasilense*	Reduction of reactive Oxygen Species (ROS) and heat shock proteins pre-activation	Abd El-Daim et al., 2014
Potato	*Paraburkholderia phytofirmans*	Reduction of H_2_O_2_ and Production of ACC.	Sangiorgio et al., 2020

### Low Temperatures

Frost events have been reported in several counties in recent years, such as in Italy, Switzerland, Germany, Belgium, USA, and France, resulting in serious crop damage and economic losses (Unterberger et al., 2018; Vitasse and Rebetez, 2018). Low temperatures decrease plant metabolism, leading to reduced photosynthetic activity, reduced foliar growth, and early senescence, when temperatures are above the freezing point (Huner et al., 1993). Bud development can be hampered by temperatures below freezing since rehydrated and growing buds might be destroyed by the cold stress (Mishra et al., 2012). Cold can dehydrate plant tissues, causing a rise in the concentration of osmolytes in the cytoplasm of the cell and leading to the breakdown of the plasma membrane (Mishra et al., 2012).

Microorganisms possessing ice nucleating activity (INA^+^), which can survive on fruits, leaves, and roots, can promote the development of ice cores in plants (Lindow, 1983). The extracellular polymeric substances (exopolysaccharides) produced by these microorganisms are made up of proteins that function as ice nucleation centers on their cell wall, promoting the production of ice crystals (Lee et al., 1995). These microbes are mostly bacteria, although ice-nucleating fungi have also been discovered (Pouleur et al., 1992), which may colonize the plant both epiphytically and endophytically. For example, *P. syringae* was the first bacterial strain reported to possess ice nucleating activity (Arny et al., 1976; Fadiji and Babalola, 2020a,b; Galambos et al., 2021). The presence of *P. syringae* strains with ice-nucleating activity enhanced the sensitivity of soybean and tomato to cold damage in experimental conditions (Anderson et al., 1982). Likewise, other species exhibited ice nucleating activity, such as *Erwinia herbicola* (syn: *P. agglomerans*) (Lindow et al., 1978), *Xanthomonas campestris* (Kim et al., 1987), and the Gram-positive bacteria *Lysinibacillus* sp. (Failor et al., 2017). On the other hand, frost damage was minimized using *P. syringae* A506 with an inactivated ice-nucleating gene (Skirvin et al., 2000) and the use of microbial biostimulants capable of competing INA^+^ bacteria has become a critical strategy for reducing frost damages (Figure 2; Selvakumar et al., 2012). For example, Blightban A506^®^ (Nufarm Americas, Inc., TX, United States), which was produced using lyophilized *P. fluorescens* A506 (Lindow and Brandl, 2003), is a commercial product for the prevention of frost damage in USA (Table 1) and this formulation is also used to combat fire blight (*E. amylovora*) in pear and apple trees (Lindow and Brandl, 2003).

Microbial biostimulants can also help to reduce the effect of chilling temperatures. Plant growth suppression caused by low temperature can be countered by microbial symbionts that produce growth-related hormones (e.g., auxins or gibberellins) or that reduce ethylene concentration (Figure 2 and Table 1; Sangiorgio et al., 2020). Several bacteria produce indole-3-acetic acid (IAA), which represents an example of auxin (Amara et al., 2015). For instance, IAA is produced by *P. dispersa* 1A and *S. marcescens* SRM strains isolated from the Himalayan northwest (Amara et al., 2015). Thus, wheat seedlings inoculated with these strains and cultivated under cold condition exhibited considerably better nutrient and yield absorption ability competed to control seedlings (Selvakumar et al., 2008a). Likewise, cold tolerance is enhanced in wheat seedlings treated with *Pseudomonas* sp. NARs9 and PGERs (Mishra et al., 2008, 2012). Cold stress causes a rise in ethylene production, which reduces plant productivity (Glick, 2014) and *P. phytofirmans* PsJN, which exhibited ACC deaminase activity, improved cold tolerance in grapevine by decreasing the damage to the cell membrane (Theocharis et al., 2012). Bean plants cultivated in extreme cold conditions and inoculated with psychrophilic ACC deaminase-producing bacteria, such as *P. fragi*, *P. fluorescens*, *P. proteolytica*, *B. frigoritolerans*, and *P. chlororaphis*, revealed less frost damage through reduced ROS production and lipid peroxidation (Tiryaki et al., 2019). Out of all the bacterial species identified, *P. fluorescens* and *P. chlororaphis* are the examples of the few microbial biostimulants found in the market as biopesticides (Cedomon^®^, BioAgri AB, Uppsala, Sweden) while the other one is formulated in combination with similar PGPR products such as HyperGalaxy^®^ (Holmes Enviro, Llc., Philomath, OR, United States), BFMS^®^ (Tainio, Cheney, WA, United States), SoilBiotic^®^ (SoilBiotics, Reddick, IL, United States) and BioStrain^®^ (Monty’s Plant Food Company, Louisville, KY, United States). More studies toward the formulation of microbial biostimulant to combat cold stress in crop plant is advocated.

### Salinity and Drought Stress

Drought or excessive salinity can induce water stress, which happens when leaf transpiration losses or surpass root absorption, resulting in a decrease in water content and turgor in plant tissues (Sangiorgio et al., 2020). Drought stress has been increasingly severe and frequent, especially in semi-arid regions (Trenberth et al., 2014). Furthermore, when rainfall is light and irregular, salt buildup in the soil can worsen the effects of drought (Othman et al., 2006), since a rise in the solutes level in the saline soil lowers the osmotic potential of the liquid phase of the soil, limiting the absorption of water by plant roots (Sequi, 2006). For example, soil salinization affects around 30% of total arable land in the Mediterranean regions (Rasool et al., 2013). Drought stress and the resulting soil salinization are also major contributors to desertification in overexploited regions, they affect soil biodiversity and composition, contributing to plant deterioration, sparse soil covering and consequent soil erosion (Vicente-Serrano et al., 2020). Drylands now occupy 46% of the world’s land surface, impacting about 250 million persons in developing countries (Huang et al., 2020).

Drought stress influences plant physiology and morphology, causing harmful ROS buildup (Smirnoff, 1993), emission of ethylene (Ali et al., 2014), and decreased mineral nutrient availability, absorption, and transport (Rouphael et al., 2012). Plant tolerance to drought and salinity can be improved by microbial biostimulants through a variety of direct and plant-mediated processes (Figure 2 and Table 2). For example, microbial biostimulants can produce bacterial exopolysaccharides that enhance soil structure by forming micro and macroaggregates (Grover et al., 2011), improving plant development under water stress conditions (Sangiorgio et al., 2020). Furthermore, exopolysaccharides form hydrophilic biofilms and create a microenvironment that promotes the retention of water by shielding microbes from drought stress (Nwodo et al., 2012) and binds Na^+^ ions to reduce uptake by the plant (Tewari and Arora, 2014). For example, *Pseudomonas* spp. PF23 (Tewari and Arora, 2014) and *Pseudomonas putida* GAP-P45 (Tallon et al., 2003) exhibit protective potentials by the production of exopolysaccharides. Exploring root capacity can be strengthened by mycorrhizal fungi (Khan et al., 2015), allowing for increased root biomass, improved structure of the soil, increased water retention, and reduced leaching of the mineral nutrients (Cavagnaro et al., 2015; Santoyo et al., 2021a). In particular, *Glomus* sp. generates a glycoprotein called glomalin, which has an aggregating impact on soil structure and improves plant development and drought tolerance (Gong et al., 2013). Likewise, ascomycetes (*P. glomerata* LWL2, *Exophiala* sp. LHL08, *P. formosus* LHL10, and *Penicillium* sp. LWL3) colonize cucumber plants, enhancing leaf development and chlorophyll content under drought stress (Khan et al., 2015; Fadiji et al., 2020). Apart from promoting root development, mycorrhizal fungi can also improve water absorption through aquaporins (Aroca et al., 2007), which is one of the large families of integral membrane transporters that enable the flow of water through the phospholipid bilayer layer of the cell membrane (Bray, 2000). For example, *Glomus intraradices* mycorrhizes common bean plant and mitigate water stress affecting aquaporin activity and improving water conductivity in the roots (Aroca et al., 2007; Omomowo et al., 2018). *Glomus intraradices*, when cultivated in association with carrot, increased the expression of two fungal aquaporins (GintAQPF2 and GintAQPF1), which consequently enhanced water transport between the 2 symbionts and therefore gave the plant higher resilience to water shortage (Li T. et al., 2013). *G. intraradices* is commonly found in commercial products, often formulated in combination with many other beneficial bacterial and fungal strains, such as OroSoil^®^ (Fomet, Spa, Verona, Italy) and MycoApply^®^ All Purpose (Mycorrhizal Applications, Inc., OR, United States), but it also exists in single formulations, such as Agtiv^®^ (PremierTech, Rivière-du-Loup, Canada) and Groundwork^®^ (GroundWork BioAg, Ltd., Hashahar, Israel).

**TABLE 2:** Microbial biostimulants that mitigate salinity and drought stress in crops.

Crop Plant	Microbes involved	Mechanism of action	References
**Protection against salinity stress**			
Tomato	*Leclercia adecarboxylata* MO1	ACC deaminase and IAA production	Khan et al., 2019
French bean	*Aneurinibacillus aneurinilyticus* and *Paenibacillus* sp.	VOCs, ACC deaminase and IAA production	Gupta and Pandey, 2019
Canola	*Pseudomonas* spp.	ACC deaminase and IAA production	Akhgar et al., 2014
Pepper	*Burkholderia and Bacillus*	Enhancement of proline accumulation	Ait Barka et al., 2006
Tomato	*Pseudomonas fluorescens* YsS6 and *Pseudomonas migulae* 8R6	IAA production	Ali et al., 2014
Soybean	*Pseudomonas simiae*	Quinoline and Glycine max 4-nitroguaiacol promote seed germination	Vaishnav et al., 2016
Tomato	*Pseudomonas fluorescens* UM270	IAA and VOCs production	Hernández-León et al., 2015
Lettuce	*Bacillus subtilis*	Cytokinin signaling and shoot biomass	Arkhipova et al., 2007
Potato	*Bacillus* spp.	IAA production	Tahir et al., 2019
**Protection against drought stress**			
Wheat	*Bacillus thuringiensis* AZP2	Production of volatile organic compounds	Khan et al., 2018
Sunflower	*Pseudomonas putida* strain GAP-P45 and *Pseudomonas* spp. PF23	Production of exopolysaccharide	Tallon et al., 2003; Tewari and Arora, 2014
Pepper	*Bacillus licheformis* strain K11	Stress-related protein and genes	Lim and Kim, 2013; Ali and Xie, 2020
Maize	*Burkholderia phytofirmans* PsJN and *Enterobacter* sp. FD	Reduced H_2_O_2_ induced damage	Naveed et al., 2014
Wheat	*Streptomyces coelicolor* DE07, *Streptomyces olivaceus* DE10, and *Streptomyces geysiriensis* DE27	Phytohormone (IAA) synthesis and increment in water stress tolerance	Yandigeri et al., 2012
Mung bean	*Staphylococcus* sp. Acb12, *Staphylococcus* sp. Acb13 and *Staphylococcus* sp. Acb14	Production of indole acetic acid, ACC deaminase, and promotion of plant growth	Jayakumar et al., 2020
Pepper	*Bacillus, Achromobacter*, *Klebsiella* sp. and *Citrobacter* sp.	Production of ACC deaminase	Marasco et al., 2012
Cucumber	*Pterolepis glomerata* LWL2, *Exophiala* sp. LHL08, *Paecilomyces formosus* LHL10, and *Penicillium* sp. LWL3	Production of glomalin	Gong et al., 2013; Khan et al., 2015
Beans and carrot	*Glomus intraradices*	Increasement in the expression aquaporins (GintAQPF2 and GintAQPF1)	Aroca et al., 2008; Li T. et al., 2013
Orange	*Funneliformis mosseae*	Production of IAA	Liu et al., 2018
Cucumber	*Acinetobacter*, *Pseudomonas*, and *Burkholderia* spp.	Production of gibberellins	Kang et al., 2015
Soybean	*Pseudomonas putida* H-2-3	Synthesis of ABA	Kang et al., 2014
Basil	*Pseudomonas* sp., *Bacillus lentus*, and *Azospirillum brasilense*	Production of Ascorbate, peroxidase and glutathione peroxidase	Heidari and Golpayegani, 2012
Wheat	*Pseudomonas libanensis* EU-LWNA-33	Adaptive phosphorus solubilization, production of Ammonia, hydrogen cyanide, and ACC deaminase.	Kour et al., 2020c
Foxtail millet	*Acinetobacter calcoaceticus EU- LRNA-72* and *Penicillium* sp. *EU-FTF-6*	Enhancement of the accumulation glycine betaine, proline, sugars, increased chlorophyll content, and decrease in lipid peroxidation	Kour et al., 2020d
Millet	*Streptomyces laurentii EU-LWT3-69* and *Penicillium* sp. *strain EU-DSF-10*	Enhancement of the accumulation of glycine betaine, proline, sugars, and decrease in lipid peroxidation	Kour et al., 2020b

Microbial biostimulants have a variety of additional effects against drought stress on associated plants, such as the antioxidant defenses, the production of protective osmolytes (e.g., glycine betaine) or phytohormones, and the secretion of volatile organic compounds (VOCs) (Figure 2 and Table 2; Kaushal and Wani, 2016; Sharifi and Ryu, 2018). Since stress condition boosts ethylene synthesis, which limits plant development, microbial biostimulants (e.g., *P. fluorescens*) might subtract ACC, reduce ethylene synthesis and relieve inhibition mediated by ethylene (Glick, 2014). Other microbial species that produce ACC in reducing drought stress include *Staphylococcus* sp. Acb12, *Staphylococcus* sp. Acb13, Staphylococcus sp. Acb14, *Bacillus, Achromobacter*, *Klebsiella*, and *Citrobacter* spp. (Marasco et al., 2012; Jayakumar et al., 2020). Beneficial microorganisms that produce ACC deaminase might help to mitigate these side effects under drought and salt stresses, such as *A. piechaudii* ARV8 in pepper and tomato plants (Mayak et al., 2004) and *P. fluorescens* TDK1 in the peanut seedlings (Saravanakumar and Samiyappan, 2007). Plant growth promotion and soil improvement are attributed to *Achromobacter* spp. and *Pseudomonas* spp. and these beneficial microorganisms are developed in commercial products, such as SSB^®^ and SOS^®^ (Liventia, Inc., San Antonio, TX, United States) (Table 3).

**TABLE 3:** Summary of some currently available commercial products used in the mitigating abiotic stresses.

Commercial name	Composition	Application	Production company	Country of production
Endox^®^	*Bacillus* spp.	Mitigation of heat stress	Scam, spa	Italy
Activate^®^	*Bacillus* spp.	Mitigation of heat stress	Natural resources Group, Inc.	United States
SoilPro^®^	*P. fluorescens* and *P. aeruginosa*	Mitigation of heat stress	Liventia	United States
Blightban A506^®^	*P. fluorescens* A506	Prevention of frost damage	Nufarm Americas, Inc.	United States
Cedomon^®^	*P. fluorescens* and *P. chlororaphis*	Reduction of frost damage	BioAgri AB, Uppsala	Sweden
HyperGalaxy^®^	*P. fluorescens* and *P. chlororaphis*	Reduction of frost damage	Holmes Enviro, Llc., Philomath	United States
BFMS^®^	–	Reduction of frost damage	Tainio, Cheney	United States
SoilBiotic^®^	–	Reduction of frost damage	SoilBiotics, Reddick, IL	United States
BioStrain^®^	–	Reduction of frost damage	Monty’s Plant Food Company	United States
OroSoil^®^		Mitigation of drought stress	Fomet, Spa, Verona	Italy
MycoApply All Purpose^®^	–	Mitigation of drought stress	Mycorrhizal Applications, Inc.	United States
Agtiv^®^	–	Mitigation of drought stress	PremierTech, Rivière-du-Loup	Canada
Groundwork^®^	–	Mitigation of drought stress	GroundWork BioAg, Ltd.	Israel
SSB^®^ and SOS^®^	*Achromobacter* spp. and *Pseudomonas* spp.	Mitigation of drought and salt stresses	Liventia, Inc.	United States
Wettable Mycorrhizae Blend^®^	*Funneliformis mosseae*	Mitigation of drought stress	BioLogic Crop Solutions, Inc.	United States
N-Texx^®^	*P. putida*	Mitigation of drought stress	CXI, Coppell	United States
Ryze^®^	–	Mitigation of drought stress	L.Gobbi, Srl	Italy
Micosat F^®^	–	Mitigation of drought stress	CCS, Srl, Aosta	Italy
Grolux^®^	–	Mitigation of drought stress	RRR Supply Inc.	United States
HyperGalaxy^®^	–	Mitigation of drought stress	Holmes Enviro, Llc.	United States
Environoc^®^	–	Mitigation of drought stress	Biodyne, Llc.	United States

Meanwhile, microbial biostimulants can synthesize IAA, which stimulates plant growth and root branching of the plant under drought stress (Ouyang et al., 2017), for example, species belonging to *Alcaligenes*, *Sinorhizobium*, *Serratia*, *Bacillus*, and *Arthrobacter* (Ouyang et al., 2017; Table 2). In particular, when inoculated on salt-stressed cucumber and tomato plants, the bacterium *P. chlororaphis* TSAU13, an IAA producing strain, can enhance plant tolerance to drought and salinity (Egamberdieva, 2012). Likewise, the mycorrhizal fungus *Funneliformis mosseae* improves IAA level in the root, root hair development, and growth of orange plants under drought stress (Liu et al., 2018). The Wettable Mycorrhizae Blend^®^ (BioLogic Crop Solutions, Inc., CA, United States) is a commercial product based on *Funneliformis mosseae* that improves the plant capacity to absorb nutrients and water (Sangiorgio et al., 2020). Similarly, microorganisms that produce cytokinins and gibberellins are effective in reducing water stress damage by promoting stomatal opening and shoot development in low-water circumstances (Liu et al., 2013). PGPR strains of *Acinetobacter*, *Pseudomonas*, and *Burkholderia* spp. have been characterized as being able to produce gibberellins and these bacteria can boost cucumber growth under salt and drought conditions (Kang et al., 2015). Despite their potentially helpful properties, no commercial products based on these species are available, suggesting that novel products will be probably launched in the future.

After exposure to drought stress, the synthesis of ABA increases to promote stomatal closure. When inoculated with *P. putida* H-2-3, ABA content decreases in soybean, reducing the impact of drought stress on plant productivity (Kang et al., 2014). *P. putida* is sold as the commercial product N-Texx^®^ (CXI, Coppell, TX, United States) in conjunction with *B. subtilis* for their roles on soil fertility, but not particularly for drought stress alleviation. Likewise, *G. intraradices* inoculation lowers ABA concentration and reduces sensitivity to salt stress in lettuce (Jahromi et al., 2008). Plants produce more ethylene because of ABA and water shortage and plant development can be reduced by high ethylene levels.

Under water stress, the production of ROS and oxidative damage to lipids, nucleic acids, and proteins are common. Many microbes can mitigate the negative effects of increased ROS by producing antioxidant molecules or increasing the activity of antioxidant plant enzymes, such as peroxidases and catalase (Vurukonda et al., 2016). When inoculated with *Pseudomonas* sp., basil plants exhibited an increment in the activity of catalase under water stress (Vurukonda et al., 2016). Likewise, *Pseudomonas* sp., *Bacillus lentus*, and *Azospirillum brasilense* are an example of microbial stimulants that have been used individually or in the formulation of microbial consortia which can reduce drought stress in crop plants (Sangiorgio et al., 2020). Ascorbate, peroxidase and glutathione peroxidase activities of *Pseudomonas* sp., *Bacillus lentus*, and *Azospirillum brasilense* was used to enhance drought stress (Heidari and Golpayegani, 2012).

Osmocompatible solute accumulation is a type of stress response that allows the accumulation of inorganic and organic solutes in the vacuole and cytosol, respectively, to lower the osmotic potential of the cell and to maintain its turgor under water stress (Sangiorgio et al., 2020). Numerous bacteria produce osmolytes (Paul et al., 2008), which often work together with osmolytes produced by the plant (e.g., proline) to lower the osmotic potential and stabilize the cell wall components (Sanders and Arndt, 2012). The phosphate-solubilizing *B. polymyxa* strain produces proline when inoculated in tomato plants, reducing the effects of water stress (Shintu and Jayaram, 2015). Betaine produced by osmotolerant bacteria from the rhizosphere (e.g., *Streptomyces tendae* F4) often works in tandem with that produced by the host plant in rice, leading to an increased tolerance to water stress (Dimkpa et al., 2009). Even though the results were encouraging, *B. polymixa* is not yet available as a commercial product.

Some bacteria can interact with plants by VOCs, which can trigger stress adaption reactions mediated by mineral uptake, water conservation, and root growth (Bailly et al., 2014). While the importance of hormone signaling cascades has been demonstrated (Bhattacharyya et al., 2015; Tahir et al., 2017), the underlying VOC-mediated mechanisms involved in plant-microbe associations under extreme conditions remain mainly unknown. Beneficial effects of microbial VOCs are known on plants, such as 2,3-butanediol on plant fitness (Sangiorgio et al., 2020), synthesis of osmoprotectants and control of stomata closure (Zhang et al., 2010). Likewise, 1-heptanol, 3-methyl-butanol, and 2-undecanone produced by *Parabulkholderia phytofirmans* enhance salinity tolerance (Ledger et al., 2016), whereas butyrolactone and 1-butanol increase the root growth and the exchange of carbon in plant’s rhizosphere (Gutiérrez-Luna et al., 2010). The future of VOC-based plant promotion will almost certainly hinge on the identification of stress-induced signaling pathways. The production of VOCs by *Bacillus thuringiensis* AZP2 helped in the mitigation of drought stress in wheat plant (Khan et al., 2018).

Although there are a large number of microorganisms that are capable of protecting plants from water stress, a limited number of commercial products exist for this vital purpose. The majority of these market products Ryze^®^ (L.Gobbi, Srl, Genova, Italy), Micosat F^®^ (CCS, Srl, Aosta, Italy; Suma), Grolux^®^ (RRR Supply Inc., Munger, MI, United States), HyperGalaxy^®^ (Holmes Enviro, Llc., Philomath, OR, United States, BFMS^®^ (Tainio, Cheney, WA, United States), Environoc^®^ (Biodyne, Llc., Wayne, IN, United States), and SoilBiotics^®^ (SoilBiotics, Reddick, IL, United States) are just a few of the successfully commercialized products based on consortia of *Azospirillum brasilense*, *Bacillus altitudinis*, *Bacillus amyloliquefaciens*, *Bacillus licheniformis*, *Cellulomonas cellasea*, *P. fluorescens*, *Pseudomonas putida*, *Pseudomonas stutzeri*, *Streptomyces albidoflavus*, *Glomus* spp., and *Trichoderma* spp., that exhibit water stress capacity, as well as enhancement of plant yield through the production of exopolysaccharides alongside the enrichment of nutrients and soil organic matter (Table 3). Efforts toward the formulation/production of more universally accepted commercial products, which will be active in most ecosystems will go a long way in the mitigation of water stress in crop plants.

### Flooding, Water Stagnation, and Heavy Rainfall

Interannual rainfall and seasonal variability patterns of rain are two of the most significant problems of the current climate change (Foster and Rahmstorf, 2011; Stevenson et al., 2012). Flooding affects 13% of the Earth’s surface, and severe rainfall intensity and frequency are expected to rise worldwide in the future (Sangiorgio et al., 2020). Water stagnation, anoxia and hypoxia of the root are caused by heavy floods and rains. The enzyme ACC synthase, noted in the production of ethylene, is produced in significant amounts by roots when they are flooded (Sangiorgio et al., 2020). The enzyme ACC oxidase, requires oxygen to catalyze the final stage of biosynthesis of ethylene and ACC is transported to the aerial portion of the plant via the xylem (Else and Jackson, 1998), where it is transformed to ethylene, resulting in wilting, chlorosis, leaf necrosis, fruit and flower loss, and limited yield (Glick, 2014). Thanks to ACC deaminase activity, which lowers the level of endogenous ethylene, microbial biostimulants can help to alleviate stress related to water stagnation (Glick, 2014; Ali and Kim, 2018). For example, tomato seedlings inoculated with ACC deaminase-producing strains of *Pseudomonas* spp. and *Enterobacter* spp. show a reduction of anoxia stress and an improvement of germination (Grichko and Glick, 2001; Sangiorgio et al., 2020). The commercial product SumaGrow^®^ (RRR Supply Inc., Munger, MI, United States), which contains *Pseudomonas* and *Enterobacter* spp., increases yield and improves stress tolerance, but it does not promise to give particular protection from waterlogging stress. Chlorophyll content, plant elongation, biomass, formation of the adventitious root, and leaf area were promoted, while ethylene levels were reduced, using *Pseudomonas* sp. on cucumber seeds (Li J. et al., 2013) and *Streptomyces* sp. GMKU 336 strain on *Vigna radiata* (Jaemsaeng et al., 2018). The active ingredients of Mycostop^®^ (Verdera Oy, Espoo, Finland) and Actinovate^®^ (Mycorrhizal Applications, Inc., OR, United States) are *S. lydicus* WYEC 108, and *Streptomyces* K61, respectively. Streptomyces spp. is beneficial bacteria frequently utilized against biotic stresses and their application against abiotic stresses is currently understudied. Future studies into the production of more microbial stimulant and the full exploration of the roles of all currently available commercial products for the mitigation of water stress is needed.

## General Constrains and Prospects in the Utilization of Microbial Biostimulants

The development of a novel microbial biostimulant presents some unique challenges. To begin with, the business registration procedure is often complicated, and there is still a lack of unified international regulation (Backer et al., 2018). Secondly, it should be noted that product effectiveness highly relies on the type of agricultural crop used, its phenological stage and environmental conditions (Khan et al., 2018). The relationship between microorganisms and host plants must be evaluated while developing microbial biostimulants. To establish the efficacy of microbial biostimulant, favorable and long-lasting colonization of the plant is required, and it could be affected by the indigenous plant-associated microbial communities (Sangiorgio et al., 2020).

Finally, to reduce the effects of cultural and environmental factors on product conservation and efficacy, the optimum formulation should be developed (Bashan et al., 2014). Due to spore formation by bacteria, biostimulants that are formulated using Gram-positive bacteria allow powder formulations to have drying tolerance and long-term stability. Even though there are several types of the effectiveness of microbe utilization in stimulating plant development under adverse conditions, there are relatively few biostimulant products available that particularly address the challenges exacerbated by climate change. Moreover, the high costs associated with microbial biostimulant registration and manufacturing (Nadeem et al., 2014) are key roadblocks to product development, resulting in a small number of products that are commercialized and limited adoption in horticulture. Thus, several factors must be taken into consideration for microbial biostimulant development and use, such as crop and soil characteristics, type of application and microbial strain characteristics. About the first aspect, no microbes can be said to be universally fit for all ecosystems (Adesemoye et al., 2009) or on any plant host (Finkel et al., 2017), indicating that selection of a strain for the production of microbial biostimulant requires consideration of the plant and the soil properties to maximize efficacy under specific conditions (Ipek et al., 2014). Biostimulant efficiency can be reduced when there is competition for space and nutrients between the selected microbial strains and the indigenous microbiota (Ruzzi and Aroca, 2015). Thus, isolation and application of endemic microbial strains, possibly with multiple plant growth promotion traits would be an important issue to observe better results in field trials (Etesami and Maheshwari, 2018). For example, it was well demonstrated that microorganisms extracted from the host plant microbiome are more effective than non-indigenous microbial inoculants (Mazzola and Freilich, 2017). Moreover, it is also important to optimize the mode of microbial biostimulant application (e.g., seed or root inoculation, spore spray, etc.), which should minimize microbe dispersion or mortality caused by abiotic variables, such as temperature and UV light effect (Vejan et al., 2016).

Crop breeding strategies in the future should include the plant ability to form permanent symbiotic partnerships with beneficial microbes as a very important feature, strongly connected to stress tolerance, resilience and production (Msimbira and Smith, 2020). Simultaneously, a better understanding of microbial activities and interaction processes may allow for the selection of appropriate microbial biostimulants for a certain cultivar or crop under a specific cultural situation (Clúa et al., 2018). Likewise, other approaches are required to fully realize the potential of microbial biostimulants, such as a depth characterization of the plant microbial biocoenosis using real-time investigation microbial functional dynamics, next-generation sequencing such as RNA/metagenome/amplicon sequencing optimization and development of the synthetic community of microbes (Fadiji and Babalola, 2020c). As a result, a critical step in the effective selection of microbial biostimulants is the characterization of the indigenous microbiome using high-throughput next-generation sequencing approaches in combination with meta-analysis of the associated population, to identify microorganisms that can persist on plants in harsh conditions (Purahong et al., 2018; Fadiji and Babalola, 2020c). Furthermore, understanding and exploiting plant-microbe interactions requires detailed research of the microbiome using functional markers rather than taxonomic markers (Lemanceau et al., 2017). The use of real-time monitoring tools for important microbial activities might be used to optimize the effectiveness of microbial inoculants. For example, the use of real-time PCR techniques enables for broad-range assessment of the functional genes available in the microbes and might be applied for future monitoring of the agroecological function (Bouffaud et al., 2018).

Finally, the creation of synthetic communities of microbes, i.e., the combination of numerous microbes with specific plant growth-promoting functions, offers an opportunity to improve the effectiveness and reliability of microbial biostimulants (Ahkami et al., 2017). However, to ensure the sustainability of the beneficial community, the intricacy of ecological interactions between microorganisms should be thoroughly explored, such as competition and commensalism.

## Conclusion and Future Prospects

Microbial biostimulants have the potential to be a long-term and successful method for reducing the abiotic stressors that climate change has exacerbated. Furthermore, the application of microbial biostimulants may help to maintain the ecological balance of agro-ecosystems, reducing the usage of pesticides and/or heavy metals for agricultural practices. Nonetheless, several concerns should be considered both at the regulatory level and throughout the development and research, to achieve greater efficacy of the product and wider adoption.

Plant biostimulant has a claim-based definition, which means that the function is used to establish the product. Many potent ingredients with various activities and objectives can be found in a single product. As a result, the inherent heterogeneity of microbial biostimulants could elude regulatory categorization (e.g., fungicide, fertilizer, and amendment). Products may be subjected to lengthy and costly trial procedures depending on the country of registration. The lack of a consistent worldwide regulatory framework creates a barrier to product marketing and may deter the development of novel products. In agroecological and biological research, the adoption of microbial biostimulants still has certain drawbacks, mostly due to their lesser efficacy and greater environmental sensitivity when compared to synthetic products (e.g., pesticides, fertilizers, and growth regulators). Furthermore, the efficacy of microbial biostimulants varied widely according to the crop and environmental conditions. Future research should focus on developing better-targeted products, such as delving deeper into interactions of the microbial biostimulant with indigenous plant-associated microbiomes.

The application of microbial biostimulants might provide a long-term and cost-effective solution to plant productivity losses caused by changing climatic factors, as well as aid in the optimization of human inputs in agro-ecosystem. Results from preliminary experiments involving microbial biostimulants should be disseminated by all relevant policymakers and stakeholders, such as extension services and growers, to ensure that this methodology can be largely applied to a variety of crops, regions, and under different environmental conditions.

## Author Contributions

AF and OB conceived the ideas, collected the data, and developed the manuscript. GS and MP provided professional input and critiqued the work. All authors have carefully read the final manuscript and have agreed that the manuscript be published.

## Conflict of Interest

The authors declare that the research was conducted in the absence of any commercial or financial relationships that could be construed as a potential conflict of interest.

## Publisher’s Note

All claims expressed in this article are solely those of the authors and do not necessarily represent those of their affiliated organizations, or those of the publisher, the editors and the reviewers. Any product that may be evaluated in this article, or claim that may be made by its manufacturer, is not guaranteed or endorsed by the publisher.
